# Enhancing Wheat Tolerance to Cadmium Stress through Moringa Leaf Extract Foliar Application

**DOI:** 10.1155/2024/2919557

**Published:** 2024-09-30

**Authors:** Muhammad Hafeez ul Rehman Khursheed, Muhammad Shahbaz, Tahrim Ramzan, Arslan Haider, Muhammad Faisal Maqsood, Arbaz Khan, Usman Zulfiqar, Muhammad Jamil, Sadam Hussain, Abdullah Ahmed Al-Ghamdi, Humaira Rizwana

**Affiliations:** ^1^ Department of Botany University of Agriculture, Faisalabad 38040, Pakistan; ^2^ Department of Botany The Islamia University of Bahawalpur, Bahawalpur 63100, Pakistan; ^3^ Department of Agronomy Faculty of Agriculture and Environment The Islamia University of Bahawalpur, Bahawalpur 63100, Pakistan; ^4^ College of Agronomy Northwest A&F University, Yangling, Xianyang 712100, China; ^5^ Department of Botany and Microbiology College of Science King Saud University, P.O. 2455, Riyadh 11451, Saudi Arabia

## Abstract

Cadmium, a hazardous heavy metal prevalent in plants and soil, poses a significant threat to human health, particularly as approximately 60% of the global population consumes wheat, which can accumulate high levels of Cd through its roots. This uptake leads to the translocation of Cd to the shoots and grains, exacerbating the potential health risks. However, promising results have been observed with the use of moringa leaf extract (MLE) foliar spray in mitigating the adverse effects of Cd stress. The current experiment was conducted to find out the Cd stress tolerance of wheat varieties V1 = Akbar-19 and V2 = Dilkash-2020 under exogenous spray of MLE. The treatments of this study were *T*0 = 0% MLE + 0 *µ*M Cd, *T*1 = 3% MLE + 0 *µ*M Cd, *T*2 = 0% MLE + 400 *µ*M Cd, and *T*3 = 3% MLE + 400 *µ*M Cd. Cd stress demonstrated a significant reduction in morphological attributes as shoot and root fresh weight (22%), shoot and root dry weight (24.5%), shoot and root length (22.5%), area of leaf and number of leaves 30.5%, and photosynthetic attributes (69.8%) in comparison with control. Exposure of wheat plants to Cd toxicity cause oxidative stress, increased H_2_O_2_, and MDA up to 75% while foliar application of MLE reduced the activities of reactive oxygen species (ROS). The activity of catalase (CAT), superoxide dismutase (SOD), peroxidase (POD), and ascorbic acid (AsA) increased up to 81.5% as well as organic osmolytes such as phenolics, total soluble proteins, and total soluble sugars were improved up to 77% by MLE applications under Cd stress. Higher accumulation of ionic contents root Na^+^ (22%) and Cd (44%) was documented in plants under Cd stress as compared to control, while uptake of root mineral ions Ca^2+^ and K^+^ was 35% more in MLE-treated plants. In crux, Cd toxicity significantly declined the growth, photosynthetic, and biochemical parameters while 3% MLE application was found effective in alleviating the Cd toxicity by improving growth and physiological parameters while declining reactive oxygen species and root Na^+^ as well as Cd uptake in wheat.

## 1. Introduction

Wheat (*Triticum aestivum* L.), a higher plant member of largest and most varied family, Poaceae, serves as a primary cereal crop. It is locally farmed worldwide and is polyploid in nature. Roughly half of the globally consumed calories come from wheat, which also possesses high reserves of macronutrients, micronutrients, dietary fiber, and vitamins B and E, as well as proteins (gluten) [1]. The storage proteins found in wheat seeds are a significant source of nutrition and energy and are crucial in determining the quality of bread that is made. Using SDS-PAGE, the two groups of wheat grain proteins gliadins and glutenins have been extensively investigated [2]. Wheat is a crop that yields well, stores well, and can be grown in a variety of climates. Human communities have chosen and refined these and other desirable traits in wheat from its most basic form to the kind that is currently farmed since ancient times [3]. The Fertile Crescent is where wheat was first domesticated about ten-thousand years ago and from there, it extended to every part of the world [4]. Wheat may be grown worldwide in temperate, tropical, and subtropical climates due to its high genetic variety. Around 25,000 distinct cultivars of wheat are cultivated across the globe [5].

Many biotic and abiotic factors can affect the wheat, but the major issue that seriously decreases wheat production is heavy metal exposure [6]. Due to shortage of water in developing nations, wastewater is commonly used to irrigate wheat, which causes a buildup of Cd stress in the grain [7]. Furthermore, research revealed that Cd is found in the root zone, from which it is translocated to shoots and other plant parts, such as grains [8]. Due to a variety of risky human activities, excessive use of synthetic fertilizers, and rock erosion, Cd is typically emitted by industry [9, 10]. The self-replicating nature of plant tissues was severely impacted by the higher levels of Cd in the root rhizosphere, which interfered with the morphological, physiological, and biochemical functions of plants [11]. Numerous limitations have been brought about by rapid industrialization, urbanization, and inadequate environmental planning. A significant drop in crop yields, particularly in wheat as well as rice [6, 12]. People in underdeveloped countries use around 80% of the wheat harvest as a staple meal, but people in developed countries use less than 50%. As a result, people in developing countries ingest a lot of Cd, which poses a serious risk to human health. In spite of health concerns, research on biotic and abiotic stress mechanisms is necessary to promote growth and productivity [13].

Regrettably, agricultural substances enhance the chance of wheat contamination, specifically with respect to Cd, and consequently elevate the probability of several inflammatory, metabolic, and chronic ailments [14]. Both natural and man-made sources can contaminate wheat, which significantly lowers overall crop production [15]. The main human factors that elevated the risk of Cd buildup in wheat are mining operations, industrial pollutants, and phosphate fertilizers. Another significant cause of Cd is industrial effluents which reaches pond used to grow wheat as well as other cereal crops [16]. Amongst cereal crops, wheat has a higher capacity to absorb Cd than other cereals. It suggests that Cd accumulation in several wheat components is harmful [13]. According to recent research, wheat absorbs Cd from its roots and subsequently translocates it into its aerial regions. High concentrations of Cd restrict the development of shoot and interfere with physiological functions including as conductance of stomata, chlorophyll production, photosynthesis, respiration, and transpiration. Cadmium translocation appears to be a quick process [17].

A number of different biological biostimulants are currently being used to increase a range of crops' production and growth. Moringa leaf extract (MLE), obtained from *Moringa oleifera* Lam. (colloquially known as moringa), is one such alternative crop used as biostimulant. Its impact on the development and production of crops under normal and stressful conditions is being investigated [18]. Significant DPPH-free radical-damaging action is accounted by MLE, which reduces osmotic damage-induced lipid peroxidation-induced cell membrane degradation [19]. Moringa leaves also contain phytohormones, including auxin, jasmonic acid, cytokinin, abscisic acid, salicylic acid, and gibberellins [20]. Foliar spray of MLE has demonstrated encouraging outcomes in mitigating the impact of several abiotic circumstances on plants, such as saline stress, low temperatures, and drought, as well as heavy metals. Cd stress had a negative impact on plant development parameters, but MLE was able to overcome these effects [21]. This was probably because Moringa leaves have a high antioxidant content, which boosted the action of enzymes such as peroxidase, ascorbate peroxidase, and catalase that work on the antioxidative system [22]. Reducing negative effects of ROS in plants induced by Cd [23] may be possible with the help of the well-known plant species *Moringa oleifera*, which has an adequate amount of fatty acids, proteins, vitamins, carbohydrates, flavonoids, and phenols [24]. In order to combat Cd toxicity, the extract of MLE is an easy to use, reasonably priced, locally produced, and environmentally acceptable substitute for inorganic fertilizers. It was proposed that using MLE topically might increase wheat tolerance to Cd stress. It was hypothesized that foliar application of MLE may enhance the Cd stress tolerance in wheat. The objectives of the study were (i) to investigate the Cd stress tolerance of wheat through foliar application of MLE and (ii) to find out the role of MLE on the production of antioxidants in wheat under Cd stress.

## 2. Materials and Methods

### 2.1. Experimental Design

The experiment was carried out at the old Botanical Garden University of Agriculture, Faisalabad. Seeds of two wheat cultivars (Akbar-2019 and Dilkash-2020) were collected from Ayyub Agricultural Research Institute. Thirty-two plastic pots, each containing 7 kg of soil, were utilized, with 16 pots allocated for each wheat cultivar. Cd solutions were prepared using Cd chloride (CdCl_2_) to induce two levels of Cd stress: control and a concentration of 400 *µ*M. Cd stress was imposed 30 days after seed sowing. Fresh leaves of *Moringa oleifera* were harvested from the Moringa Germplasm Unit, University of Agriculture, Faisalabad, and brought to the Botany lab for further processing. These leaves were submerged in ice for 12 hours and then subjected to maceration using a 96% ethanol solvent extraction technique. Approximately 300 grams of powdered moringa leaf were steeped in 1,700 milliliters of solvent in a glass container, covered with aluminum foil, and left to soak for three days with periodic stirring. The resulting maserate was filtered using a cotton cloth, and the residue was then resoaked in 1,200 ml of fresh solvent for an additional two days. The resulting maserate was evaporated using a water bath until a thick consistency was achieved. One gram of moringa leaf extract and ten milliliters of 96% ethanol were poured to a beaker glass, which was then swirled until the ethanol was dissolved and filtered. If there are flavonoid compounds present, 1 mL of the filtrate should be placed in a test tube, 10% NaOH reagent should be added, and the color should turn orange or yellow. A 100% MLE was prepared as stock and then prepared 3% concentrations of MLE working solution from that stock solution. Two levels of moringa leaf extract were used (control and 3% MLE). Application of MLE was applied after one week of Cd stress application. For foliar spray, 30 ml of 3% MLE were applied to plants while watering to each pot via spraying shower. The experiment was conducted by using three-factor factorial under completely randomized design (CRD) with three replicates.

### 2.2. Harvesting and Data Collection

All plants were carefully uprooted to collect data, and their roots were placed in a Na2-EDTA solution followed by deionized water rinses to remove contaminants. The fresh and dry weight of the wheat was measured using a weighing balance. Plant shoot and root lengths were measured using a measuring tape. In addition, the total number of leaves per plant and the leaf area (in cm^2^) were recorded. After a week of storage at 65°C in an oven, the dry weights of the plants' roots and shoots were determined.

### 2.3. Determination of Photosynthetic Pigments

Arnon's method [25] was employed to determine the following parameters: carotenoid contents, total chlorophyll, *a/b* ratio, and chlorophyll *a*, *b*. One milligram of wheat sample was placed into small plastic bottles, followed by the addition of 5 ml of 80% acetone. After allowing the mixture to stand overnight, it was filtered using Whatman paper. The following day, a small amount of the sample was transferred to a cuvette, and absorbance measurements were taken at 663 nm, 645 nm, and 480 nm using a spectrophotometer (model IRMECO U2020). The obtained optical density (OD) values were then used with corresponding formulas to determine the concentrations of photosynthetic pigments.

(1) $$\begin{array}{c} \text{Chl }a\text{ contents}=\frac{\left(0.0127\times A663-0.00269\times A645\right)\times 100}{0.5},\\ \text{Chl }b\text{ contents}=\frac{\left(0.0229\times A645-0.00468\times A663\right)\times 100}{0.5},\\ \text{Total chlorophyll}=\text{chlorophyll }a+\text{chlorophyll }b,\\ \text{Total carotenoids}=\frac{A^{\text{car}}}{\text{Em}}\times 100,\\ \text{where Em}=2500. \end{array}$$

### 2.4. Oxidative Stress Determinants

The quantity of hydrogen peroxide was determined using the methodology described by Velikova et al. [26]. Wheat leaf extracts weighing 0.25 g were mixed with 5 milliliters of 0.1% trichloroacetic acid and centrifuged for 15 minutes at 12,000 rpm. One milliliter of the filtrate was then mixed with 0.5 mL of potassium phosphate (KH_2_PO_4_) buffer and 1 mL of KI. Subsequently, the absorbance at 390 nm was measured using a spectrophotometer (model IRMECO U2020).

MDA data were calculated using the technique outlined by Heath and Packer [27]. In brief, wheat leaf samples weighing 0.25 g were ground in 0.1% trichloroacetic acid and centrifuged for 15 minutes at 12,000 rpm. Then, 2 mL of 0.5% TBA in 20% TCA and 1 mL of supernatant were combined and incubated at 95°C. The reaction was stopped after 30 minutes of incubation by placing the mixture in an ice bath. Absorbance was ultimately measured at 600 nm and 532 nm following centrifugation at 12,000 rpm for 10 minutes at 4°C [27].

### 2.5. Enzymatic Antioxidants

The concentration of catalase in fresh wheat leaves was tested using the method outlined by Aebi [28]. Wheat leaf material was weighed, and a solution consisting of 50 mM phosphate buffer (pH 7.8) and 100 mM H_2_O_2_ was heated to 25°C in a water bath. After adding 0.2 mL of phosphate buffer and enzyme sample to a 10 mL tube, it was heated in a water bath for three minutes. The tube was then filled with 0.3 mL of the 100 mM H_2_O_2_ solution. To denature the enzyme solution, the control tube was heated for five minutes in a boiling water bath. Following mixing, absorbance was determined every minute at a wavelength of 240 nm.

For the assessment of SOD and POD activity, fresh wheat leaf samples (250 mg) were crushed in a chilled mortar and pestle. 5 mL of potassium phosphate buffer were added to each sample during grinding. After thorough mixing, the mixture was transferred to Eppendorf tubes and centrifuged for 15 minutes at 12,000 rpm. The resulting solution was transferred to additional separate Eppendorf tubes and stored at −15°C after precipitation.

Superoxide dismutase activity was determined using the method described by Spitz and Oberley [29]. Plastic cuvettes were placed under a fluorescent lamp for 15 minutes after adding 0.4 mL of D·H_2_O, 0.05 mL of leaf extract, 250 *μ*L of chilled potassium phosphate buffer, 0.05 mL of NBT (nitro blue tetrazolium) solution, 0.05 mL of riboflavin solution, 0.1 mL of triton X solution, and 0.1 mL of L-meth solution. A blank sample without a plant sample was also conducted. Absorbance of all plant samples was measured at 560 nm using a UV-visible spectrophotometer.

The POD concentration was determined following the protocol developed by Chance and Maehly [30]. The cuvette was filled with the following solutions in order: 50 *μ*L of plant extract, 750 *μ*L of potassium phosphate buffer, 0.1 mL of hydrogen peroxide, and 0.1 mL of guaiacol. Absorbance was noted at a wavelength of 470 nm at intervals of 0, 30, 60, and 90 seconds using a spectrophotometer (IRMECO U2020).

### 2.6. Ascorbic Acid

The amount of AsA in fresh wheat leaf material was measured following the method described by Yin et al. [31]. A 0.5 g leaf sample, cooled in an ice bath, was crushed using a pestle and mortar with the addition of 5 mL of 5% trichloroacetic acid. The resulting sample was transferred to Eppendorf tubes and centrifuged at 12,000 rpm for 20 minutes at 4°C. The pure sample without filtrate was used to determine the amount of ascorbate, while the remaining extract was utilized for measuring ascorbate content. In each test tube, 2 mL of the supernatant and 1 mL of 2% dinitrophenyl hydrazine were added, along with one drop of 10% thiourea dissolved in 70% ethanol. The sample extracts were centrifuged for fifteen minutes. Subsequently, 5 mL of 80% H_2_SO_4_ were added to each test tube, and the absorbance data of leaf ascorbic acids were recorded at 530 nm using the standard curve on the IRMECO U2020 spectrophotometer.

### 2.7. Determination of Phenolic Contents

The phenolic concentration was analyzed using the method developed by Julkunen-Tiitto [32]. Fresh wheat plant samples with known weights were homogenized using 80% acetone. After centrifugation at 1000 × *g* for 15 minutes, 2 mL of H_2_O, 1 mL of Folin–Ciocalteau's phenol reagent, and 0.1 mL of supernatant were thoroughly combined in a cuvette. Subsequently, 10 mL of distilled water and 2 mL of 20% Na_2_CO_3_ were added to the mixture. Absorbance was measured at 750 nm using a spectrophotometer (IRMECO U2020).

### 2.8. Determination of Total Soluble Sugar

The protocol described by Yoshida et al. [33] was utilized to determine the amount of total soluble sugar (TSS). Initially, 0.1 g of fresh plant material was added to 5 mL of distilled water (d·H_2_O). The solution was then heated and filtered through filter paper, followed by dilution up to 10 mL. Subsequently, 5 mL of the anthrone reagent was added to the reaction mixture, which was then heated for 20 minutes at 90°C. Absorption was finally detected at 620 nm.

### 2.9. Determination of Ionic Contents (mg/g DW)

Wheat plant samples were dried in an oven at 65°C for 14 days after sowing (DAS), following which each sample was finely powdered. To each flask containing 0.1 g of powdered material, 3 mL of H_2_SO_4_ was added. Two to three drops of hydrogen peroxide were then added to each flask, which were subsequently placed on a hot plate until the solution turned translucent. After cooling and filtration, distilled water (d·H_2_O) was added to bring the volume up to 50 milliliters. The levels of Ca^2+^, Na^+^, and K^+^ were measured following the method outlined by Allen et al. [34]. Concentrations of Ca^2+^, Na^+^, and K^+^ were determined using a flame photometer (Sherwood model, 410 UK). In addition, the Cd content was analyzed using a spectrophotometer with atomic absorption (PerkinElmer, Waltham, MA, USA) using the same sample.

### 2.10. Statistical Analysis

The experiment's data were analyzed using Statistix 8.1 (Analytical Software, USA) employing the analysis of variance technique (ANOVA) under the completely randomized design (CRD). Mean differences were assessed using Tukey's honestly significant difference (HSD) test [35]. Data were calculated, correlated, and graphically represented using Microsoft Excel (2016). The R software (version R-4.3.3) was utilized to generate heatmaps, while Originpro 2022 was employed for the correlation matrix.

## 3. Results

Results have exposed that a significant effect was observed on growth, physiobiochemical, and inorganic ion contents of wheat cultivars under Cd stress.

### 3.1. Morphological Attributes

The results of the statistical analysis revealed that Cd stress significantly reduced the morphological attributes of both wheat cultivars. A significant reduction (*p* < 0.05) was observed in shoot fresh weight and root fresh weight, with decreases of up to 28.6% and 24% in V1 (Akbar-19) and 23.1% and 13% in V2 (Dikash-2020) under Cd stress compared to T2 (0% MLE + 400 *µ*M Cd). The concentration of Cd stress (400 *µ*M) also led to declines in shoot dry weight and root dry weight by 20.3% and 14% and 30.3% and 34% in Akbar-19 and Dilkash-2020, respectively. In addition, shoot length decreased by 20% and 24% and root length declined by 23% and 22% in V1 (Akbar-19) and V2 (Dilkash-2020), respectively. Leaf area and the number of leaves of both wheat cultivars decreased by 30% in V1 (Akbar-19) and by 26% and 36% in V2 (Dilkash-2020), respectively.

Moreover, a significant enhancement was observed with the exogenous application of MLE as a foliar spray on the growth attributes of both cultivars compared to untreated plants. The foliar spray of MLE (3%) improved the fresh and dry weight of shoots by 12% and 11% in V1 (Akbar-19) and by 11% and 14% in V2 (Dilkash-2020), respectively. It also enhanced root fresh and dry weight by an additional 8% and 4% in V1 (Akbar-19) compared to V2 (Dilkash-2020). Shoot length increased by 12%, while root length was boosted up to 17% in both wheat cultivars with the application of MLE. The 3% level of moringa extract enhanced leaf area and the number of leaves by up to 18% and 50% in V1 (Akbar-19) and by 17% and 43% in V2 (Dilkash-2020), respectively (Table 1).

### 3.2. Photosynthetic Parameters

Cd stress has an adverse impact on the photosynthetic pigments of wheat compared to control conditions. However, foliar treatment with moringa extract significantly enhanced photosynthetic pigments, including chlorophyll *a* and *b*, total chlorophyll, and carotenoids compared to T2 (0% MLE + 400 *µ*M Cd). Notably, significant interactions were observed among wheat cultivars for photosynthetic pigments.

Chlorophyll *a* decreased significantly by 27% and 24%, chlorophyll *b* by 23% and 34%, total chlorophyll by 25% and 29%, chlorophyll *a/b* ratio by 4% and 1.9%, and carotenoid content reduced by 20% and 22% in V1 (Akbar-19) and V2 under 400 *µ*M Cd stress compared to the control. However, the results demonstrated that treatment with 3% moringa leaf extract substantially increased chlorophyll *a* by up to 19% and 13%, chlorophyll *b* by up to 17% and 23%, total chlorophyll by 18% and 17%, and carotenoid content by up to 14% and 19% in V1 (Akbar-19) and V2 (Dilkash-2020), respectively, compared to the control. Maximum enhancement in photosynthetic pigments in wheat cultivars subjected to Cd stress was noted with exogenous applications of 3% MLE (Table 2).

### 3.3. Oxidants and Antioxidants

The recorded data revealed that the activity and indicators of ROS increased under Cd stress, while foliar treatment with moringa leaf extract reduced ROS, mitigating oxidative damage in plants. Hydrogen peroxide levels increased by up to 30% and 20%, while malondialdehyde (MDA) content increased by up to 63% and 69% in V1 (Akbar-19) and V2, respectively, under Cd stress (400 *µ*M). However, the foliar spray of moringa extract reduced H_2_O_2_ content by 8% and 10%, while malondialdehyde decreased by 16% and 13% in V1 (Akbar-19) and V2, respectively.

**Figure 1:**
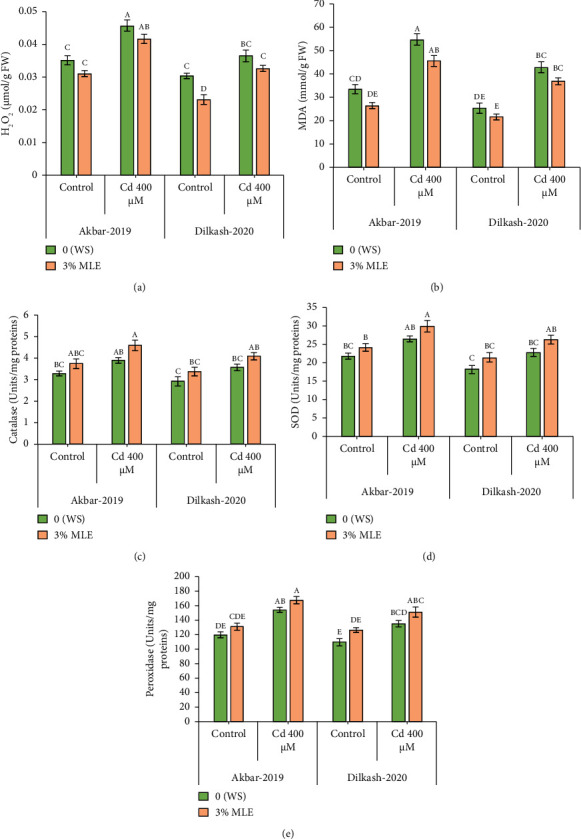
Effect of Cd and MLE on (a) hydrogen peroxide (*µ*mol g^−1^ F.W), (b) malondialdehyde (*µ*mol g^−1^ F.W), (c) catalase (units mg^−1^ protein), (d) superoxide dismutase (Units mg^−1^ protein), (e) peroxidase (Units mg^−1^ protein) of wheat. Error bars above means specify the ±SE of four replicates. Same letter sharing by means for a parameter do not vary significantly at *p* ≤ 0.05. V1 = Akbar-19; V2 = Dilkash-2020; *T*0 = 0% MLE + 0 *µ*M Cd, *T*1 = 3% MLE + 0 *µ*M Cd, *T*2 = 0% MLE + 400 *µ*M Cd, *T*3 = 3% MLE + 400 *µ*M Cd.

Under heavy metal stress (400 *µ*M Cd), enzymatic antioxidant activity, including catalase (CAT), superoxide dismutase (SOD), and peroxidase (POD), increased by up to 18%, 22%, and 21% and 25%, 28%, and 23%, respectively, in V1 (Akbar-19) and V2 compared to T2 (0% MLE + 400 *µ*M Cd). In addition, nonenzymatic antioxidant activity, such as ascorbic acid (AsA), substantially increased by 38% and 43% in V1 (Akbar-19) and V2, respectively, compared to the control. Moreover, exogenous application of moringa leaf extract improved antioxidant activity under Cd stress. Specifically, catalase, superoxide dismutase, peroxidase, and ascorbic acid activity increased by 17%, 12%, 8%, and 15% in V1 (Akbar-19) and by 14.4%, 14.8%, 11%, and 17% in V2 (Dilkash-2020), respectively (Figure 1).

### 3.4. Organic Osmolytes

**Figure 2:**
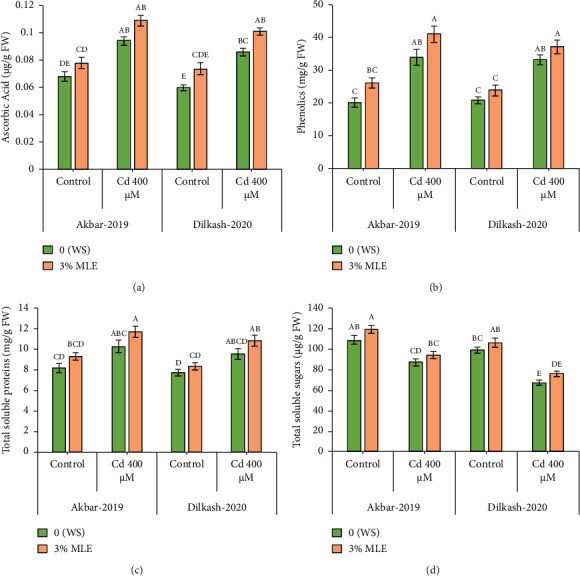
Effect of Cd and MLE on (a) ascorbic acid (*µ*g·g^−1^ FW), (b) phenolics (mg^−1^ FW), (c) total soluble sugars (*µ*g·g^−1^ FW), and (d) total soluble proteins (mg·g^−1^ protein) of wheat. Error bars above means specify the ±SE of four replicates. Same letter sharing by means for a parameter do not vary significantly at *p* ≤ 0.05. V1 = Akbar-19; V2 = Dilkash-2020; *T*0 = 0% MLE + 0 *µ*M Cd, *T*1 = 3% MLE + 0 *µ*M Cd, *T*2 = 0% MLE + 400 *µ*M Cd, *T*3 = 3% MLE + 400 *µ*M Cd.

The statistical data revealed that Cd stress (400 *µ*M) increased the concentration of osmolytes, with the activity of phenolics enhancing by 69% in V1 (Akbar-19) and 58% in V2. Similarly, total soluble protein (TSP) content increased by 25% in V1 and 23% in V2, respectively. However, the amount of total soluble sugars decreased under Cd stress by 20% in V1 and 31% in V2. The collected data also showed that the amount of total phenolics and total soluble proteins increased in both cultivars under the toxic stress of Cd compared to control conditions. In addition, exogenous treatment with MLE further improved the activity of phenolic content by 20% and 11%, while total soluble proteins enhanced by 13% in both cultivars, respectively, under Cd stress conditions. Furthermore, the exogenous application of MLE (3%) increased the total soluble sugars by up to 8% and 12% among both cultivars, correspondingly, relative to T2 (0% MLE + 400 *µ*M Cd) (Figure 2).

### 3.5. Inorganic Ions

**Figure 3:**
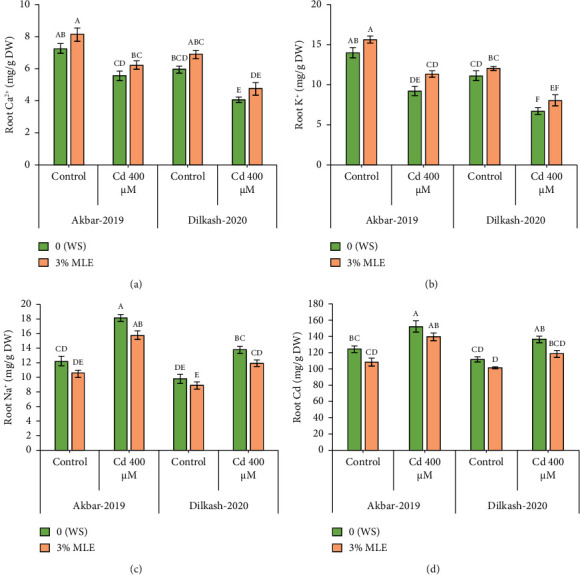
Effect of Cd and MLE on (a) root calcium (mg·g^−1^ DW), (b) root potassium (mg·g^−1^ DW), (c) root sodium (mg·g^−1^ DW), and (d) root Cd (mg·g^−1^·g DW) of wheat. Error bars above means specify the ±SE of four replicates. Same letter sharing by means for a parameter do not vary significantly at *p* ≤ 0.05. V1 = Akbar-19; V2 = Dilkash-2020; *T*0 = 0% MLE + 0 *µ*M Cd, *T*1 = 3% MLE + 0 *µ*M Cd, *T*2 = 0% MLE + 400 *µ*M Cd, *T*3 = 3% MLE + 400 *µ*M Cd.

Cd stress exposure in wheat plants led to a reduction in the uptake of mineral ions. Specifically, root Ca^2+^ ion uptake decreased by 23% and 31% in V1 (Akbar-19) and V2, respectively, while root K^+^ ion contents decreased by 34% and 39%, respectively. Moreover, Cd toxicity at a level of 400 *µ*M increased the uptake of Cd through roots by 22% and also enhanced Na^+^ ion uptake by roots up to 48% and 40% in V1 (Akbar-19) and V2, respectively. MLE at a concentration of 3% restored the loss of Ca^2+^ and K^+^ by increasing uptake through roots by up to 12% and 22% in V1 (Akbar-19) and by 16% and 20% in V2, respectively. In addition, MLE mitigated the toxic levels of Cd and Na^+^ ions in both cultivars, reducing them by 8% and 12% in V1 (Akbar-19) and by 13% in V2 (Dilkash-2020), under T2 (0% MLE + 400 *µ*M Cd) (Figure 3).

### 3.6. Heatmap with Dendrogam

**Figure 4:**
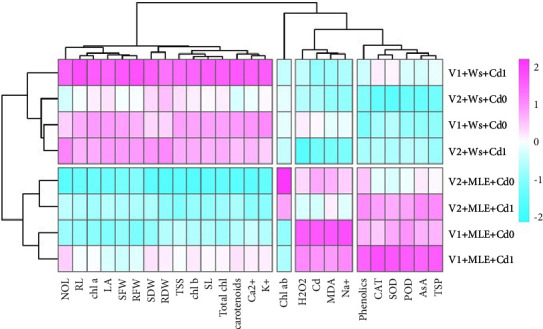
Heatmap with dendrogram between morphophysiological, biochemical attributes and ionic contents of wheat (Akbar-19 and Dilkash-2020) varieties. SFW: shoot fresh weight, RFW: root fresh weight, SL: shoot length, RL: root length, SDW: shoot dry weight, RDW: root dry weight, LA: leaf area, NOL: number of leaves, Chl. *a*: chlorophyll *a*, Chl. *b*: chlorophyll *b*, Total Chl.: total chlorophyll, Chl. *a*/*b*: chlorophyll *a*/*b* ratio, carotenoids, H_2_O_2_: hydrogen peroxide, MDA: malondialdehyde, CAT: catalase, SOD: superoxide dismutase, POD: peroxidase, AsA: ascorbic acid, Total phenolics, TSP: total soluble protein, TSS: total soluble sugar, root Na^+^: root sodium, Cd root Cd. root K^+^: root potassium, root Ca^2+^: root calcium. V1 = Akbar-19; V2 = Dilkash-2020; *T*0 = 0% MLE + 0 *µ*M Cd, *T*1 = 3% MLE + 0 *µ*M Cd, *T*2 = 0% MLE + 400 *µ*M Cd, *T*3 = 3% MLE + 400 *µ*M Cd.

Heatmap analysis along with dendrogram showed the effect of MLE used topically on various morphological, biochemical, and physiological attributes of wheat cultivars under Cd stress. Heatmap has clustered the growth attributes (shoot and root fresh and dry weight, shoot and root length, and number and area of leaves), photosynthetic attributes (carotenoids, total chlorophyll, and chl. *a*, *b*) and mineral ions such as Ca^2+^ and K^+^ in first group. Heatmap demonstrated the cluster of increasing attributes of both wheat cultivars under MLE treatment and Cd stress such as CAT, AsA, POD, TSP, and phenolic content in the fourth group. These attributes, i.e., H_2_O_2,_ MDA root Na^+^, and Cd are clustered in the third group that disclosed a strong positive correlation while Chlorophyll *a*/*b* ratio was clustered in second group under Cd stress with application of MLE (Figure 4).

### 3.7. Correlation Matrix Analysis

**Figure 5:**
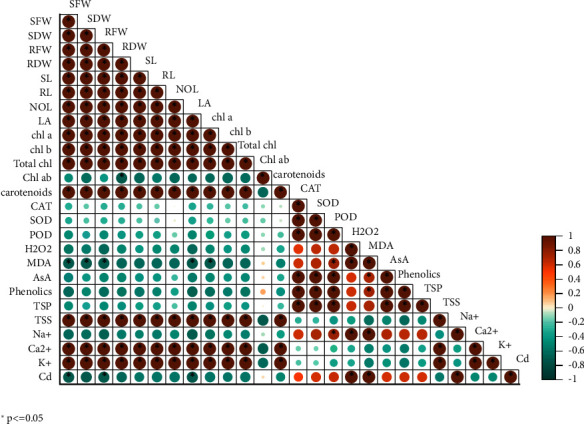
Correlation analysis between morphophysiological, biochemical attributes and ionic contents of wheat (Akbar-19 and Dilkash-2020) varieties. ^∗^*p* ≤ 0.05.

A Pearson correlation matrix analysis revealed the connections between the various parameters of wheat cultivars that were grown under Cd stress with foliar spray of MLE. Various morphological characteristics including (SFW, RFW), (SL, RL), and (SDW, RDW) as well number and area of leaves are positively correlated in all directories with pigments used in photosynthesis, such as chlorophyll *a* and *b*, total chl., and carotenoids, as well as inorganic ions such as, root Ca^2+^, and K^+^ while morphological parameters comprising SL, RL, LA, SFW, RFW, SDW, and RDW, and no. of leaves are negatively correlated with antioxidants (CAT, SOD, POD, and AsA) as well as root Na^+^ and Cd ions in all indices of wheat cultivars while grown under Cd stress (Figure 5).

## 4. Discussion

Among the most hazardous metals that have accumulated in many crops through industrial and food production is Cd. The morphophysiological and biochemical processes of wheat are negatively impacted by Cd, which indirectly affects the plant's growth [36]. One potential strategy for reducing the toxicity of heavy metals is to minimize their absorption in wheat. In the current study, we observed that using leaf extract from *Moringa oleifera* not only improved wheat growth during development but also had positive impacts on biological and ecological features in all growing environments (Figures 1, 2, 3, and 4; Tables 1 and 2).

**Figure 6:**
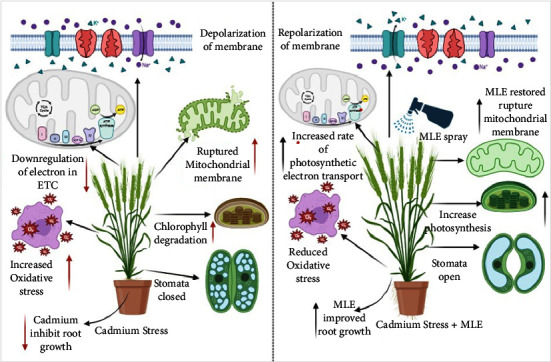
A schematic diagram represents two different mechanisms of wheat under Cd stress (control and 400 *µ*M Cd) with foliar application of MLE.

Ensuring wheat plants' reduced toxicity to Cd is crucial for maintaining food safety [37]. Growth attributes play a vital role in determining crop health and vigor. Various heavy metals, including Cd, can alter plant morphophysiological and biochemical pathways, indirectly affecting plant growth [38–40] (Figure 6). It is widely accepted that exposure to heavy metals can diminish plant growth and physiological traits [36]. Cd stress in wheat leaves also accelerates the degradation of chlorophyll pigments [41], which is consistent with our findings and previous research. The use of moringa leaf extract significantly enhanced the fresh weight, dry weight, and leaf growth of the shoots [42]. Another study [43] demonstrated that MLE acts as a biostimulant to improve wheat growth characteristics. MLE contains compounds such as auxin, zeatin, galactinol, beta-sitosterol, and cytokinin, which promote cell growth, elongation, division, biochemical processes, and yield regulation in wheat [44]. With its high concentration of growth-promoting compounds like zeatin, MLE directly contributes to improving wheat's growth-related characteristics [6]. Previous research has shown that foliar application of MLE can boost plant growth [42], and when combined with growth-promoting chemicals, it enhances plant germination parameters and seedling performance [45]. Chlorophyll fluorescence properties serve as valuable markers of growth, physiological response, and PSII modulation in plants under biotic and abiotic stress. Our data revealed a clear connection between fluorescence characteristics and growth, as all Cd levels affected both growth and chlorophyll fluorescence properties (Table 1).

According to research conducted by Yotsova and colleagues [46], compromised PSII reaction centers were observed alongside decreased chlorophyll contents. The substantial drop in Fv/Fm ratios also suggested a decrease in the qP value, possibly due to restricted stomatal openings, which limit the amount of light energy converted to chemical energy [47]. Previous studies have shown that Cd stress in wheat seedlings downregulates gas exchange characteristics [45]. Furthermore, Cd weakens the chlorophyll machinery, leading to a significant loss of photosynthetic pigments [48]. The primary reason for the decline in growth and related features is the inhibition of photosynthetic properties by Cd, including decreased chlorophyll levels, transpiration, and gas exchange [49].

Cadmium toxicity resulted in a significant reduction of chlorophyll pigments in both wheat varieties in the current study. This decline in chlorophyll pigments may have been influenced by a decrease in the activity of certain enzymes, such as peroxidase and chlorophyllase (Figure 6) [50]. According to Amirjani [51], the application of an extract made from moringa leaves enhanced the concentration of both chlorophyll *a* and *b* in the leaves of wheat. They also noted that cytokinin, a primary component of MLE, stimulates chlorophyll production in wheat. In another study, Gao et al. [47] suggested that treating wheat with MLE increased chlorophyll concentrations and may have a protective effect against the decline in photochemical efficiency caused by Cd toxicity. Our findings are in line with this data, as the administration of MLE reduced the decrease in photosynthetic pigments induced by Cd. The application of MLE induced changes in the leaf pigments of wheat, which were associated with the enrichment of zeatin in MLE. Zeatin-enriched MLE plays a crucial role in cytokinin biosynthesis, thereby preventing early leaf senescence by increasing chlorophyll levels and promoting greater accessibility to the dynamic photosynthetic leaf region [52]. Our findings align with those regarding the decline in photochemical efficiency due to Cd toxicity and the potential projection properties of photosynthesis associated with topically applied MLE [53] (Table 2).

Enzymatic antioxidants and other self-defense mechanisms play a crucial role in shielding crop plants from oxidative damage caused by external stresses [54]. Alharby et al. [55] have suggested that elevated levels of Cd contamination may induce oxidative damage, including H_2_O_2_ accumulation, ionic leakage, and lipid peroxidation. These events may subsequently trigger the activation of antioxidative enzymes, nonenzymatic antioxidants, and the expression of enzyme genes. Reactive oxygen species (ROS), such as superoxide, H_2_O_2_, and oxygen free radicals, are generated in the chloroplasts during water deficit conditions exacerbated by increased heavy metal concentrations [56]. Hence, enzymes such as ascorbate peroxidase (AsA), superoxide dismutase (SOD), catalase (CAT), and peroxidase (POD), which convert H_2_O_2_ into water and oxygen, constitute part of the antioxidative defense against ROS damage [50]. Quenchers with low molecular weight and antioxidant enzymes are crucial for determining a plant's ability to tolerate stress. According to Khan et al. [57], conditions of abiotic stress elevate H_2_O_2_ production, thereby leading to a reduction in growth-related characteristics (Figure 6).

However, excessive H_2_O_2_ synthesis causes more membrane damage and less turgidity in the membranes, which inhibits plant development. Without interfering with the plant's regular metabolic processes, the build-up and manufacture of these bioactive, and low molecular weight organic osmolytes that function as shielding agents to stop protein deterioration and cell structure breaking [58]. Several metabolic intermediates play vital roles in plant development, contributing to Cd tolerance by supporting cell wall formation, enhancing the antioxidant defense system, and regulating the metabolism of phytochelatins along with other organic ligands [59]. According to recent reports, the content of phytohormones in moringa leaves varies with the season and is linked to variations in day duration and temperature [60]. Because of this quality, moringa leaf extract are useful to protect wheat and other plants from abiotic stressors.

In addition, Cd stress raised the plant metabolism's metabolic pools that reduce ROS, hydrogen peroxide (H_2_O_2_), and malondialdehyde (MDA). According to Alharby et al. [55], the buildup of ROS species suppresses wheat's oxidative stress-promoting antioxidant enzymes. Applying a moringa leaf extract induced the triggering of antioxidant enzymes including peroxidases and catalases. In addition, they showed that the antioxidative enzymes counteracted the Cd stress in wheat by scavenging ROS. According to a different study, treating wheat with MLE increased the formation of secondary metabolites. These substances' hydroxyl and carboxyl groups attach to Cd and prevent oxidative stress [59]. However, AsA, an antioxidant enzyme found in MLE, is a source of lowering oxidative impairment and regulating multiple aspects of cell growth, such as senescence, division, and differentiation [61]. Furthermore, the use of moringa leaf extract raised amounts of amino acids and TSP in wheat that reduce Cd stress [62]. An aliphatic amino acid called proline helped wheat under stress of Cd. A current study found that MLE application was useful in reducing the Cd level through osmolyte accumulation causes and contains proteins and flavonoids that are essential for plant growth [6]. These findings are consistent with our findings (Figure 3).

The results demonstrated that in contrast to the control group that did not experience any Cd stress, both wheat types' absorption of plant nutrients, such as Ca^2+^ and K^+^, was decreased by Cd stress. Several plants' mineral nutrient contents were adversely affected by Cd stress [63]. In both wheat varieties, the shoot, leaf, and roots were decreased in the absorption of sodium, potassium, calcium, and phosphorus; however, the largest drop was noted in Dilkash-2020. Plants micro- and macronutrients rapidly reduced under Cd stress in a variety of plants, particularly wheat [64, 65]. The use of 2.5% MLE decreased the ROS-driven oxidative destruction. The mechanism to promote abiotic stress resistance in wheat was connected to the detoxification of ROS and enhanced activity of antioxidants because of the function of phenolic as a H^+^ donor singlet oxygen quencher [52].

In the present experiment, the addition of Cd to the growing medium greatly raised the concentration of Cd in both genotypes. The accumulation of Cd in plant tissue showed a fluctuating pattern in both genotypes of wheat. Contradictory results were found in Dilkash-2019 wheat variety, which showed less Cd transported in the stem and leaves and more Cd buildup in the roots. The varying distributions of metal deposition in the root, stem, and leaf may be the cause of this [66]. It is hypothesized that variations in the cell walls' capacity to bind Cd inside the root zone account for variations in Cd absorption and transport various forms. It is commonly recognized that the role of Cd cell wall binding for root cells is to block its translocation through the root xylem into the stem and leaves [67]. Cd exclusion from the protoplast may be one of several plants' mechanisms for protecting against metal stress, as the majority of the accumulated Cd was conserved by the roots in the apoplastic environment far from the cells [68] (Figure 4).

## 5. Conclusion

Cadmium stress toxicity reduced the growth, physiological, and biochemical attributes of wheat varieties resulting in reduced growth and nutrient uptake by plants. However, exogenous applications of MLE enhanced plant growth attributes by increasing the rate of photosynthetic pigments production and improving the biochemical characteristics of plants. Furthermore, MLE treatment boosted the uptake of mineral ions such as Ca^2+^ and K^+^ and increased the activities of osmolytes such as phenolics, TSP, and TSS, as well as enzymatic and nonenzymatic antioxidants such as CAT, SOD, POD, and AsA. The maximum increment was observed at 3% MLE foliar application. This treatment improves plant physiological characteristics and chlorophyll pigments, making them more resistant to drought stress. Further analysis of MLE applications at the molecular level needs to be conducted among wheat cultivars under Cd-stressed conditions.

## Figures and Tables

**Table 1:** Interactive effect of MLE and Cd stress application on morphological attributes of wheat cultivars.

Varieties	Treatments	Shoot length (cm)	Root length (cm)	Shoot fresh weight (g)	Root fresh weight (g)	Shoot dry weight (g)	Root dry weight (g)	No. of leaves	Leaf area (cm^2^)
V1	T0	44.58 ± 0.12ab	7.43 ± 0.21ab	1.99 ± 0.07ab	1.31 ± 0.05abc	0.39 ± 0.01c	0.18 ± 0.01abc	5.75 ± 0.47ab	38.67 ± 1.61ab
	T1	48.95 ± 1.59a	8.66 ± 0.44a	2.27 ± 0.08a	1.50 ± 0.06a	0.47 ± 0.008b	0.21 ± 0.01a	7.25 ± 0.25a	42.59 ± 2.07a
	T2	35.3 ± 1.71cd	5.67 ± 0.30cd	1.42 ± 0.07cd	0.98 ± 0.05d	0.31 ± 0.01d	0.15 ± 0.008bcd	4 ± 0.40bc	26.99 ± 2.18cd
	T3	39.35 ± 1.20bc	6.63 ± 0.32bc	1.59 ± 0.068cd	1.15 ± 0.04bcd	0.38 ± 0.01bc	0.17 ± 0.008abc	5.75 ± 0.47ab	31.99 ± 2.04bcd

V2	T0	39.4 ± 1.32bc	6.61 ± 0.30bc	1.71 ± 0.06bc	1.19 ± 0.04bcd	0.39 ± 0.01bc	0.19 ± 0.004ab	4.75 ± 0.47bc	34.76 ± 0.92abc
	T1	43.00 ± 1.73ab	7.39 ± 0.31ab	2.01 ± 0.07ab	1.35 ± 0.05ab	0.43 ± 0.01ab	0.20 ± 0.006a	6.75 ± 0.25a	38.66 ± 1.26ab
	T2	29.66 ± 0.99d	5.1 ± 0.34d	1.31 ± 0.05d	0.99 ± 0.02d	0.27 ± 0.006d	0.12 ± 0.006d	3 ± 0c	25.51 ± 1.23d
	T3	33.48 ± 1.51cd	5.99 ± 0.19bcd	1.50 ± 0.05cd	1.07 ± 0.06cd	0.33 ± 0.01cd	0.14 ± 0.01cd	4.5 ± 0.28bc	30.03 ± 2.42cd

Values represent the means ± standard error of four replicates. Same letter sharing by means for a parameter indicate that they do not vary significantly based on Tuckey's test *α* = 0.05. V1 = Akbar-19; V2 = Dilkash-2020; *T*0 = 0% MLE + 0 *µ*M Cd, *T*1 = 3% MLE + 0 *µ*M Cd, *T*2 = 0% MLE + 400 *µ*M Cd, *T*3 = 3% MLE + 400 *µ*M Cd.

**Table 2:** Interactive effect of MLE and Cd stress application on photosynthetic parameters of wheat cultivars.

Varieties	Treatments	Chl. *a* (mg/g FW)	Chl. *b* (mg/g FW)	Total chl. (mg/g FW)	Chl. *a*/*b* (mg/g FW)	Carotenoids (mg/g FW)
V1	T0	0.52 ± 0.01ab	0.42 ± 0.01ab	0.95 ± 0.35ab	1.23 ± 0.009b	0.065 ± 0.002ab
	T1	0.58 ± 0.02a	0.48 ± 0.02a	1.06 ± 0.05a	1.21 ± 0.021b	0.073 ± 0.002a
	T2	0.38 ± 0.01cd	0.32 ± 0.01cd	0.71 ± 0.021cd	1.17 ± 0.05b	0.05 ± 0.002bcd
	T3	0.45 ± 0.34bc	0.38 ± 0.02bc	0.84 ± 0.05bc	1.19 ± 0.06b	0.059 ± 0.003abc

V2	T0	0.47 ± 0.01bc	0.38 ± 0.01bc	0.85 ± 0.02bc	1.23 ± 0.007b	0.055 ± 0.003bcd
	T1	0.51 ± 0.01ab	0.42 ± 0.01ab	0.93 ± 0.02ab	1.21 ± 0.005b	0.062 ± 0.003abc
	T2	0.35 ± 0.02d	0.24 ± 0.02d	0.60 ± 0.04d	1.43 ± 0.038a	0.043 ± 0.003d
	T3	0.40 ± 0.01cd	0.30 ± 0.02cd	0.70 ± 0.03cd	1.31 ± 0.049ab	0.05 ± 0.0028cd

Values represent the means ± standard error of four replicates. Same letter sharing by means for a parameter indicate that they do not vary significantly based on Tuckey test *α* = 0.05. V1 = Akbar-19; V2 = Dilkash-2020; *T*0 = 0% MLE + 0 *µ*M Cd, *T*1 = 3% MLE + 0 *µ*M Cd, *T*2 = 0% MLE + 400 *µ*M Cd, *T*3 = 3% MLE + 400 *µ*M Cd.

## Data Availability

The data that support the findings of this study are available on request from the corresponding author. The data are not publicly available due to privacy or ethical restrictions.
